# Selective Brain Cooling Reduces Motor Deficits Induced by Combined Traumatic Brain Injury, Hypoxemia and Hemorrhagic Shock

**DOI:** 10.3389/fneur.2018.00612

**Published:** 2018-08-03

**Authors:** Lai Yee Leung, Katherine Cardiff, Xiaofang Yang, Bernard Srambical Wilfred, Janice Gilsdorf, Deborah Shear

**Affiliations:** ^1^Brain Trauma Neuroprotection and Neurorestoration Branch, Center for Military Psychiatry and Neuroscience, Walter Reed Army Institute of Research, Silver Spring, MD, United States; ^2^Department of Surgery, Uniformed Services University of the Health Sciences, Bethesda, MD, United States

**Keywords:** traumatic brain injury, polytrauma, selective brain cooling, neurobehavior, neuroinflammation

## Abstract

Selective brain cooling (SBC) can potentially maximize the neuroprotective benefits of hypothermia for traumatic brain injury (TBI) patients without the complications of whole body cooling. We have previously developed a method that involved extraluminal cooling of common carotid arteries, and demonstrated the feasibility, safety and efficacy for treating isolated TBI in rats. The present study evaluated the neuroprotective effects of 4-h SBC in a rat model of penetrating ballistic-like brain injury (PBBI) combined with hypoxemic and hypotensive insults (polytrauma). Rats were randomly assigned into two groups: PBBI+polytrauma without SBC (PHH) and PBBI+polytrauma with SBC treatment (PHH+SBC). All animals received unilateral PBBI, followed by 30-min hypoxemia (fraction of inspired oxygen = 0.1) and then 30-min hemorrhagic hypotension (mean arterial pressure = 40 mmHg). Fluid resuscitation was given immediately following hypotension. SBC was initiated 15 min after fluid resuscitation and brain temperature was maintained at 32–33°C (core temperature at ~36.5°C) for 4 h under isoflurane anesthesia. The PHH group received the same procedures minus the cooling. At 7, 10, and 21 days post-injury, motor function was assessed using the rotarod task. Cognitive function was assessed using the Morris water maze at 13–17 days post-injury. At 21 days post-injury, blood samples were collected and the animals were transcardially perfused for subsequent histological analyses. SBC transiently augmented cardiovascular function, as indicated by the increase in mean arterial pressure and heart rate during cooling. Significant improvement in motor functions were detected in SBC-treated polytrauma animals at 7, 10, and 21 days post-injury compared to the control group (*p* < 0.05). However, no significant beneficial effects were detected on cognitive measures following SBC treatment in the polytrauma animals. In addition, the blood serum and plasma levels of cytokines interleukin-1 and −10 were comparable between the two groups. Histological results also did not reveal any between-group differences in subacute neurodegeneration and astrocyte/ microglial activation. In summary, 4-h SBC delivered through extraluminal cooling of the common carotid arteries effectively ameliorated motor deficits induced by PBBI and polytrauma. Improving cognitive function or mitigating subacute neurodegeneration and neuroinflammation might require a different cooling regimen such as extended cooling, a slow rewarming period and a lower temperature.

## Introduction

Traumatic brain injury (TBI) often presents acute care and treatment challenges. Extending the time window for treating reparable injuries such as hemorrhage or peripheral organ damage, while maintaining cerebral viability, could be a lifesaving strategy. One of the promising approaches is selective brain cooling (SBC), in which the brain temperature is reduced by 2–3°C while maintaining normothermia throughout the rest of the body (1). This approach captures the neuroprotective benefits of hypothermia while limiting any systemic complications attributed to whole body cooling.

Therapeutic hypothermia has been shown to be neuroprotective in experimental TBI (2, 3). Preclinical studies using rodent models of focal or diffuse TBI demonstrated that post-traumatic hypothermia reduced mortality, lesion volume and neuronal damage. It also attenuated axonal damage, blood-brain barrier disruption and neuroinflammation, as well as improved behavioral outcomes following isolated TBI (4–7). Systemic cooling (whole body cooling) which is commonly used in clinical practice for other indications (e.g., cardiac arrest or neonatal encephalopathy), was used to deliver hypothermia in these studies. However, its potential adverse effects such as coagulopathy, hypotension and pneumonia (8, 9) may limit its use in treating TBI casualties with multiple injuries. Only a few studies examined the effects of therapeutic hypothermia in experimental TBI models complicated by secondary insults. Yamamoto et al reported that a 1-h systemic cooling (targeted brain temperature = 30°C) mitigated neuronal damage at 24 h following closed head injury with concomitant hypoxia and hypotension in rats (10). Another study showed that a 4-h local cerebral hypothermia (targeted brain temperature = 33°C) with slow rewarming reduced cortical contusion produced by combined fluid percussion injury and hypoxemic insult (11). On the other hand, a similar cooling paradigm did not improve behavioral deficits or lesion volume in a rat model of controlled cortical impact and hypoxemia (12). Incorporation of secondary insults in TBI models more closely recreates the complex pathophysiology of polytrauma. This multifaceted approach may increase the likelihood of transferring therapeutic interventions such as SBC from bench to bedside (13).

Different strategies have been developed to selectively cool the brain but none have been successfully translated into clinical practice. Ice packs around the head/neck region (1, 12) or blowing cooled air directly onto the skull (7, 11, 14–16) have been used in rodent studies. A recent study used a cooling probe placed over the cranietomized area in mice (17). Nevertheless, heat loss through conduction or convection might not be sufficient to effectively cool the subcortical or deep brain structures. In addition, the cooling probe cannot be used in the presence of wound or skull fractures. Endovascular infusion of ice-cold fluid has been tested in non-human primate using a micro-catheter placed in the proximal middle cerebral artery. It was shown to be effective in lowering the brain temperature within 10 min to 33.9°C and maintaining for 20 min (18). Yet, a longer cooling duration requires more fluid infusion, which may exacerbate intracerebral hemorrhage in TBI patients.

We have previously established a selective brain cooling method in rats that involved extraluminal cooling of the common carotid arteries using bilateral cooling cuffs. This method effectively reduces the brain temperature (cortical and subcortical) within 30 min without complications (19, 20). In a rat model of cerebral ischemia, reductions in infarct size and peri-infarct depolarization were evident following 90-min of cooling (19). More importantly, protection against intracerebral hemorrhage progression, elevated intracranial pressure, brain edema, impaired blood-brain barrier permeability, lesion size and neurological deficits have been demonstrated following 2-h cooling in a rat model of severe penetrating TBI (20, 21). We further investigated the effects of treatment onset or duration, and found that brain cooling delayed by 2–4 h or extended by 4–8 h still achieved multiple beneficial effects similar to the 2 h of cooling initiated immediately after severe TBI in rats (22). However, it is unclear whether these neuroprotective benefits can be reproduced when TBI is complicated by other insults. The current study focused on evaluating the neuroprotective effects of 4-h brain cooling initiated immediately following injury in a rat model of severe penetrating TBI combined with hypoxemic and hypotensive insults.

## Methods

### Subjects

Male adult Sprague-Dawley rats (300–330 g; Charles River Labs, Raleigh, NC, USA) were used in these experiments. All procedures involving animal use were reviewed and approved by the Institutional Animal Care and Use Committee (IACUC) of Walter Reed Army Institute of Research. Research was conducted in compliance with the Animal Welfare Act and other federal statutes and regulations relating to animals and experiments involving animals and adheres to principles stated in the Guide for the Care and Use of Laboratory Animals, NRC Publication, 2011 edition. Animals were housed individually under a 12 h light/dark cycle in a facility accredited by the Association for Assessment and Accreditation of Laboratory Animal Care International (AAALACI).

### PBBI, HX, and HS

Anesthesia was induced with 3.5% isoflurane delivered in air/oxygen mixture [Fraction of inspired oxygen (FiO_2_) = 0.26] and maintained at 1.5% throughout the surgery. In all animals, the right femoral artery and vein were cannulated for mean arterial pressure (MAP) monitoring and fluid resuscitation, respectively. In addition, the tail artery was cannulated for inducing HS by withdrawing blood. Rats were randomly divided into 2 groups that included the control group (PHH; *n* = 13) and the treatment group (PHH+SBC groups; *n* = 12). Both groups received unilateral (right) PBBI that was induced using a simulated ballistic injury device (Mitre Corp., McLean, VA) with a specially designed stainless steel probe (Popper & Sons Inc., Hyde Park, NY). The probe was mounted to a stereotaxic arm at an angle of 50° from the vertical axis and 25° counter-clockwise from the anterior-posterior axis. It was then, manually inserted through the right frontal cortex of the anesthetized rat via a cranial window (+4.5 mm A-P, +2 mm M-L from Bregma) to a distance of 12 mm (from dura). The elastic tubing on the probe was inflated by a rapid (<40 ms) water pressure pulse, forming an elliptical balloon calibrated to 5% of the total rat brain volume to produce an intracerebral temporary cavity. The probe was then gently retracted and the cranial opening was sealed with sterile bone wax. Transient HX was induced 5 min after PBBI by reducing FiO_2_ to 0.1 (10% oxygen balanced with 90% nitrogen), resulting in a PaO_2_ of < 40 mmHg. Normoxia (FiO_2_ = 0.26) was restored after 30 min of HX. Five minutes following restoration of normoxia, transient HS was induced by withdrawing blood via the tail arterial catheter using a withdrawal pump (Harvard Apparatus, Holliston, MA) at a constant rate of 0.25 ml/100 g/min to reduce MAP to 30–45 mmHg (monitored via femoral artery catheter connecting to a blood pressure transducer). HS was maintained for 30 min before receiving fluid resuscitation with lactated Ringer's solution (Hospira, Lake Forest, IL) via the femoral vein catheter. The infusion volume was three times the blood volume withdrawn.

### Selective brain cooling (SBC)

Animals were randomly assigned to one of the two groups: PHH+SBC (*n* = 12) and PHH (without SBC; *n* = 13). Five minutes following fluid resuscitation, SBC was induced by using cooling cuffs around the common carotid artery (CCA) as described previously (19). The cuffs were placed around the exposed segment of each CCA (~0.5 cm below the bifurcation of internal and external carotid artery) and secured by a piece of silk suture. Brain temperature was monitored via a temperature probe inserted into the left cerebral hemisphere. Core body temperature was monitored using a rectal temperature probe (Harvard Apparatus, Holliston, MA) and maintained at ~37°C using a heating blanket. Ice-cold water was pumped into the cuffs continuously to cool the arterial blood as it entered the brain. The target brain temperature was 2–3°C lower than the baseline measurement. Spontaneous rewarming was achieved by termination of cold-water circulation. The total cooling time was 4 h. All physiological data (body and brain temperatures, MAP and heart rate) were acquired using PowerLab data acquisition system and analyzed using LabChart software (ADInstruments, Colorado Springs, MO). PHH group underwent the same procedures (same duration of anesthesia) without the cooling.

### Rotarod task

Motor coordination and balance were evaluated on Rotamex-5 rotarod apparatus (Columbus Instruments, OH). Prior to any surgical procedures, rats were trained for 4 days to meet the criterion of maintaining walking balance on the rotarod for >45 s at 3 fixed-speed increments (10, 15, and 20 rpm). Rats did not meet the criterion on the fifth day were excluded from the study. Baseline performance at these three speeds was recorded on the day prior to injury. Two trials were performed at each speed. Rats were tested again at 7, 10, and 21 days post-injury using the same parameters as defined for pre-injury baseline measures. Mean latency to fall off the rotarod at each speed was recorded. This task was performed by an investigator blinded to the groups.

### Morris water maze (MWM) task

Cognitive abilities were assessed using a spatial learning paradigm of the MWM (Noldus EthoVision XT, VA). The MWM apparatus consists of a circular basin (75 cm deep; 175 cm diameter) filled with clear water (22°C room temperature) to a depth of 60 cm placed in a dark room with visual cues. A clear, plexiglas platform was submerged to a depth of 2.5 cm from the water surface and placed in the center of the northwest quadrant of the pool. The platform position remained constant throughout all experiments. Rats were placed in the pool at one of the equally spaced starting positions (north, south, east, and west). The starting position was pseudo-randomly determined for each trial within a day, alternating between short- and long-arms in reference to the platform. Each rat was allowed to swim freely to find the hidden platform or until 90 s elapsed. Rats were given 4 trials per day (30 min inter-trial interval) for 5 consecutive days, from 13 to 17 days post-injury. A probe trial (missing platform test) was given on the last day (post-injury day 17) following the last trial of the spatial learning test. Each rat was allowed to swim freely until 60 s elapsed. Mean latency to find the hidden platform and time spent in the target zone searching for the missing platform (probe trial) were recorded. This task was performed by an investigator blinded to the groups.

### Histology

On 21 days post-injury (the last day of MWM task), animals were transcardially perfused with phosphate-buffered saline (PBS), followed by 4% cold paraformaldehyde under deep anesthesia. Coronal brain sections (40 μm) were cut from +3.72 to −6.84 mm anteroposterior from Bregma. Four sets of serial sections were collected at 960-μm intervals. All the samples were processed at FD NeuroTechnologies (Ellicott City, MD). The first set was processed for the detection of neurodegeneration with FD NeuroSilver™ Kit II (FD Neurotechnologies, Ellicott City, MD) according to the manufacturer's instructions. The second and third sets of sections were immunostained for astrocyte [glial fibrillary acid protein (GFAP)] and microglia (ionized calcium-binding adaptor molecule 1) respectively. Briefly, after inactivating the endogenous peroxidase activity with hydrogen peroxidase, sections were incubated free-floating in 0.01 M PBS (pH 7.2) 1% normal donkey serum (Jackson ImmunoResearch, West Grove, PA), 0.3% Triton X-100 (Sigma, St. Louis, MO) and the specific antibodies (rabbit anti-GFAP – 1:15,000, Dako/Agilent Technologies, Santa Clara, CA; rabbit anti-Iba-1 – 1:8,000, Wako Chemicals, Richmond, VA) for 24 h at 4°C. The sections were then incubated for 2 h at room temperature with biotin conjugated secondary antibody and detected by Vectastin elite ABC kit (Vector Lab, Burlingame, CA) and 3′,3′-diaminobenzidine (Sigma, St. Louis, MO) as a chromogen. Subsequently, all stained sections were mounted on microscope slides and cover-slipped with Permount (Fisher Scientific, Fair Lawn, NJ). Images of the sections were digitized using an Olympus VS120 Whole Slide Scanning System (Olympus Corporation of the Americas, Waltham, MA) at uniform criteria for sensitivity and exposure time. For GFAP, Iba-1 and silver staining, positive-stained cells were quantified using threshold analysis in the cerebral cortex, hippocampus or corpus callosum (silver staining only). The threshold value was set to consistently detect maximal positive staining of GFAP or Iba-1 or silver. To ensure objective quantification, the same threshold value was applied to all brain sections for each respective marker. All quantification was performed by an investigator blinded to the groups using Image J (NIH, Version 1.49).

### Enzyme-linked immunosorbent assays (ELISA)

Blood was collected 21 days post-injury by cardiac puncture using Z/1.3 clotting tubes (serum; Sarstedt, Newton, NC) or using heparin-coated microcentrifuge tubes (plasma) and allowed to clot at room temperature (serum) or on ice (plasma) for 30 min before centrifugation at 1,200 g for 10 min at 4°C. Serum and plasma were transferred to a storage tube and kept in −80°C until subsequent analyses. Interleukin-1β (IL-1β) and −10 (IL-10) sandwich ELISAs were conducted using commercially available kits according to the manufacturer's protocol (Thermo Fisher Scientific, Waltham, MA). Signal intensity was measured in duplicate using a colorimetric plate reader at 450 nm and 550 nm. Target protein concentration was determined from standards. Detection and accuracy were confirmed with internal calibrator controls.

### Statistical analysis

Statistical analysis was performed using SAS software v9.1 (SAS Institute, NC) and SigmaPlot 12.0 (Systat Software Inc., CA). Two-way repeated measures analysis of variance (ANOVA) was used to analyze the physiology and behavioral data. Pairwise *post-hoc* analysis was performed with the Student-Newman-Keuls (S-N-K) test. One-way ANOVA was used to compare the data obtained from the probe trial of Morris water maze. Student's *t*-test was used to determine the between-group difference in histology and ELISA data. The significance criterion of all the statistical tests was set at *p* < 0.05. Data are presented as the mean ± standard error of means (SEM).

## Results

### General physiology

**Figure 1:**
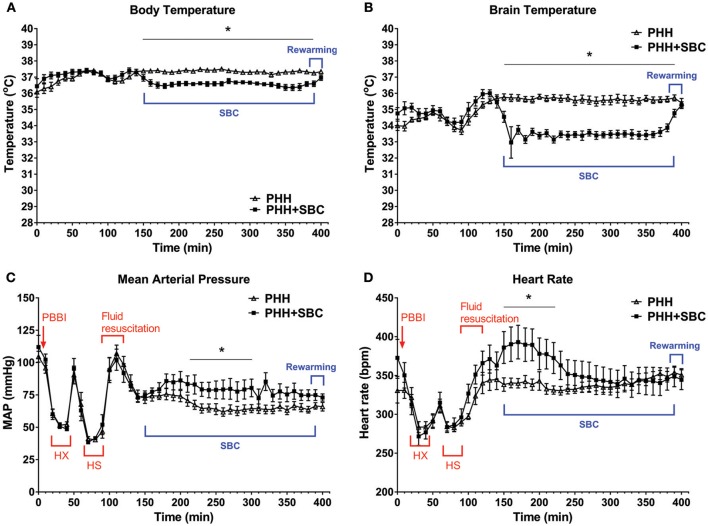
Acute physiological parameters. Body temperature **(A)**, brain temperature **(B)**, mean arterial pressure **(C)**, and heart rate **(D)** were monitored continuously from pre-injury baseline to the end of rewarming. Data was analyzed as 2-min averages taken at 5-min intervals across the 400-min recording period. For better visualization, data was plotted every 10 min on these graphs. PHH group *n* = 13; PHH+SBC group *n* = 12. ^*^p < 0.05 (two-way repeated ANOVA with S-N-K *post-hoc* test).

Baseline body temperature in both groups was maintained between 36~37°. It dropped about 1° in the SBC group throughout the cooling period (Figure 1A). Brain temperature of the SBC group dropped 2~3° within 30 min, indicating an effective cooling to the targeted temperature. During the spontaneous rewarming period, the brain temperature in the SBC group rose from 33 to 35° within 30 min (Figure 1B).

As shown in Figures 1C,D, MAP and heart rate in both groups dropped significantly during the hypoxemic and hypotensive states but they returned to baseline levels following fluid resuscitation. The MAP then gradually decreased to <80 mmHg and remained low in the control group (PHH) throughout the recording period. In contrast, SBC increased the MAP to about 90 mmHg, which was maintained at a level that was significantly higher than the control group halfway through the cooling period (*p* = 0.013–0.047). On the other hand, heart rate in the SBC group increased significantly only during the initial phase of brain cooling (*p* = 0.002–0.047; two-way repeated ANOVA with S-N-K *post-hoc* test).

### Rotarod task

**Figure 2:**
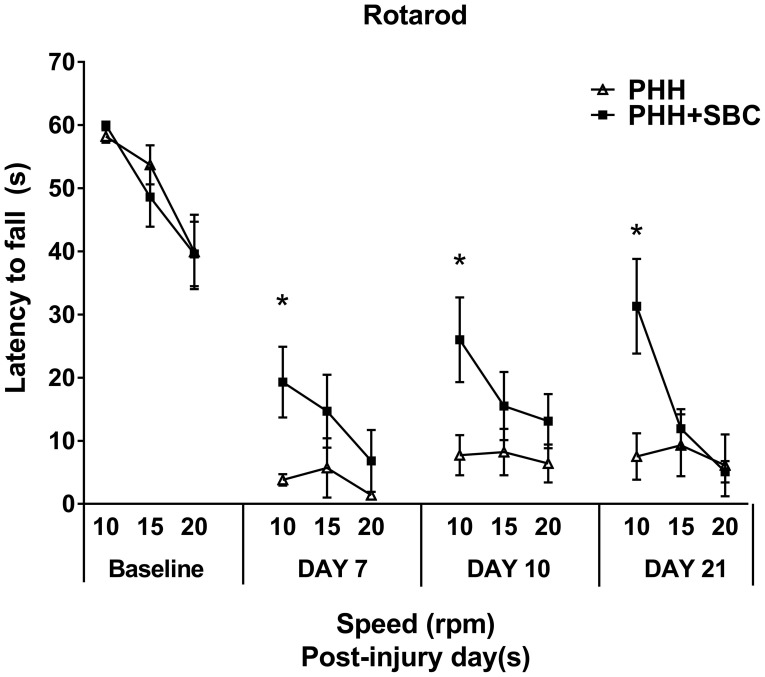
Motor function assessed by the latency to fall in the rotarod task at the pre-injury baseline, 7, 10, and 21 days post-injury. PHH group *n* = 13; PHH+SBC group *n* = 12. ^*^p < 0.05 (two-way repeated ANOVA with S-N-K *post-hoc* test).

Prior to injury, all groups showed similar baseline levels of motor performance on the rotarod task (Figure 2). Significant and persistent motor deficits were observed from day 7 to 21 in both groups. However, animals that received SBC performed significantly better (longer latency to fall) than the animals without SBC at the speed of 15 rpm on day 7, 10, and 21 days post-injury (*p* = 0.015, 0.008, and 0.001 respectively; two-way repeated ANOVA with S-N-K *post-hoc* test).

### Morris water maze task

**Figure 3:**
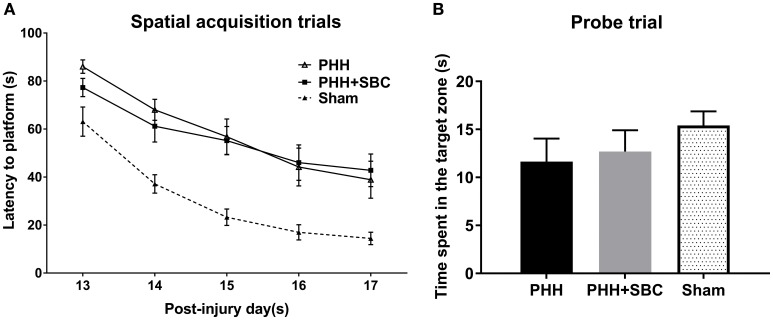
Cognitive function assessed by the latency to platform in the spatial learning task **(A)** and time spent in the target zone in the probe trial **(B)** using MWM at 2 weeks post-injury. The data of sham control group was from our previous study and used as a reference to show the cognitive deficits on the injured animals. PHH group *n* = 13; PHH+SBC group *n* = 12. Sham control group *n* = 12. Two-way repeated ANOVA and one-way ANOVA detected no significant difference between groups.

Spatial learning performance was assessed in all groups at 13–17 days post-injury (Figure 3A). The results showed that combined PBBI and HH insults produced significant cognitive deficits as evidenced by the increased latency to platform compared to our previous data obtained from sham control animals. A trend toward improved cognitive function was observed in the SBC group on day 13, yet no significant differences were detected in either the acquisition or the probe (missing platform) trials (Figure 3B).

### Histology

**Figure 4:**
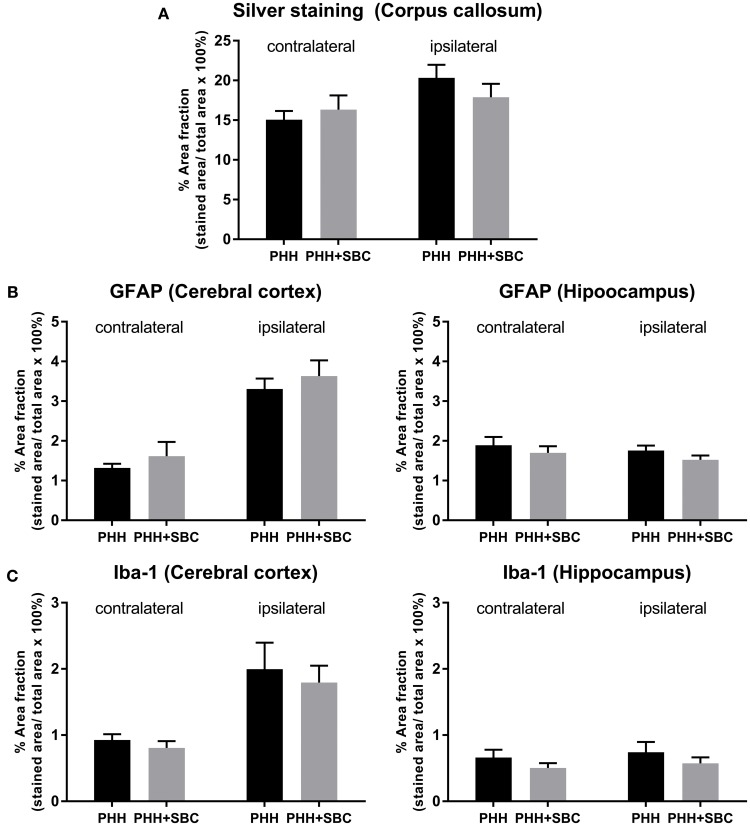
Quantification of neurodegeneration by silver staining in the corpus callosum **(A)**, astrocytic activation by GFAP **(B)** and microglial activation by Iba-1 **(C)** in the cerebral cortex and hippocampus of both hemispheres. PHH group *n* = 13; PHH+SBC group *n* = 12. Student's *t*-test did not detect statistically significant differences between groups.

In both groups of animals, PHH resulted in substantial neurodegeneration (15–20% of the corpus callosum area), indicated by the silver staining (Figure 4A). Increased GFAP (Figure 4B) and Iba-1 (Figure 4C) immunoreactivities were detected in the ipsilateral cerebral cortices of both groups, but not the hippocampi. However, none of these markers showed significant differences between the PHH and PHH+SBC groups at 21 days post-injury.

### ELISA

**Figure 5:**
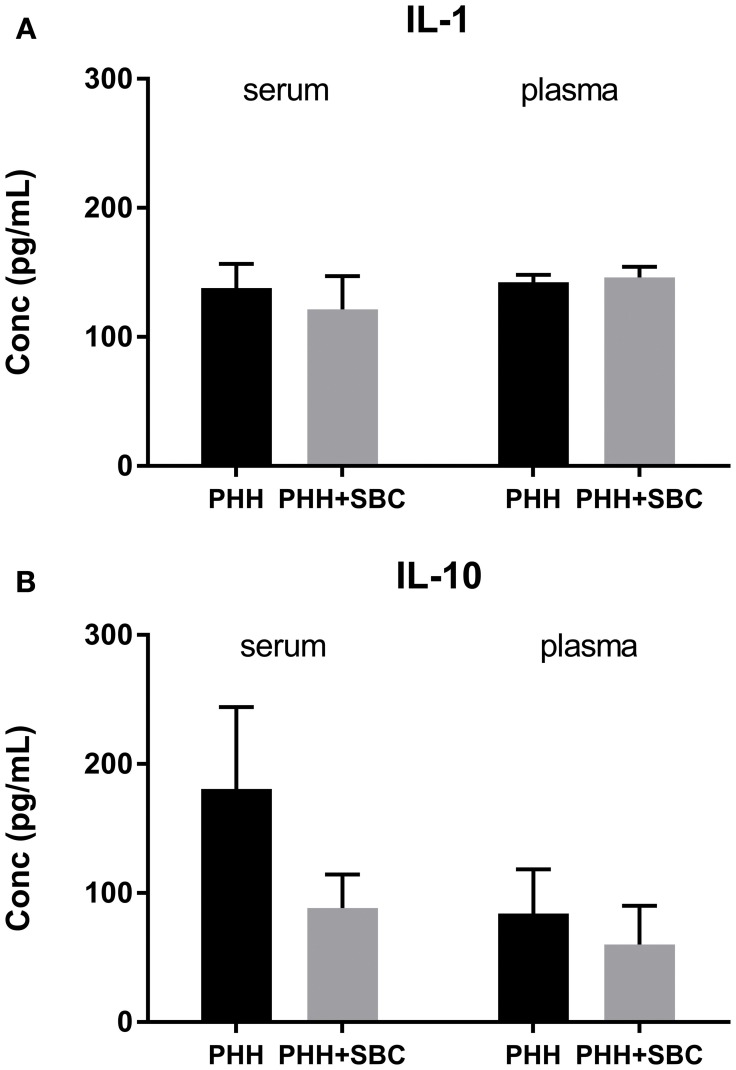
Blood serum and plasma levels of IL-1 **(A)** and IL-10 **(B)**. PHH group *n* = 13; PHH+SBC group *n* = 12. Student's *t*-test did not detect statistically significant differences between groups.

As shown in Figure 5, the plasma levels of IL-1β and IL-10, as well as the serum IL-1β were comparable between the two groups at 21 days post-injury. In PHH group, the serum level of IL-10 trended higher than that in the PHH+SBC group, but did not reach statistical significance (*p* = 0.20; Student's *t*-test).

## Discussion

Our current study showed that 4-h SBC delivered through extraluminal cooling of the common carotid arteries effectively ameliorated motor deficits induced by combined PBBI, hypoxemic and hypotensive insults. The beneficial effects persisted for a prolonged period of time. In addition, SBC transiently augmented cardiovascular function, as indicated by the increase in MAP and HR during cooling. Yet, the treatment did not mitigate cognitive deficits, sub-acute neurodegeneration or neuroinflammation.

The efficacy of SBC for improving motor function was consistent with our previous study in which the improvement was sustained through 21 days following 4 or 8-h SBC in animals subjected to isolated PBBI (22). Additional hypoxemic and/or hypotensive insults have been shown to worsen the cerebral hemodynamics (low cerebral blood flow and brain oxygenation) acutely following PBBI (23), as well as the neurobehavior during the subacute phase (24). In the present study, cerebral perfusion was likely increased by the augmented cardiovascular function (transient increase in mean arterial pressure and heart rate) during the cooling period. Our previous study showed that PBBI resulted in an increase of ICP to about 15 mmHg at 1 h post-injury (25). While the MAP in the control group and the treatment group was 60 and 75 mmHg, respectively during the cooling period, the cerebral perfusion pressure (CPP) should be around 45 mmHg in the control group and 60 mmHg in the treatment group. It was suggested that a CPP of 60 mmHg provides adequate perfusion for most TBI patients (26). Lower CPP leads to reduction in cerebral blood flow and is frequently associated with unfavorable outcomes. On the other hand, higher CPP worsens cerebral edema and intracranial hypertension (27). Thus, maintaining cerebral perfusion within an optimal range is critical for minimizing secondary injury following acute brain injury. Without using fluid or vasopressors, SBC using the extraluminal method was able to increase the MAP and possibly CPP during the cooling period in the PBBI animals. This might, in part, contribute to the improved behavioral outcome. More importantly, SBC ameliorated the outcomes worsened by additional insults following TBI, indicative of its strong and robust neuroprotective effects.

No significant improvement in cognitive function (acquisition and retention) was detected in animals treated with 4-h SBC after combined PBBI, hypoxemia and hemorrhagic shock. This is consistent with our previous studies using the isolated PBBI model (20, 22). In fact, extending the SBC duration to 8 h still did not mitigate the cognitive deficit induced by PBBI alone (22). Other preclinical TBI studies showed mixed results. Improved cognitive outcomes were reported by (6) and (28) after hypothermia treatment, with temperatures lower than the present study (32°C for 2 h in controlled cortical impact (CCI) model and 30°C for 3 h in fluid percussion injury model, respectively). In contrast, no effect on cognitive function was detected in the study using 4-h hypothermia treatment at 32°C following CCI and hypoxemic insult (12). Factors such as types and levels of injury, therapeutic window of the treatment, cooling duration and rewarming rate were suggested to determine the efficacy of the hypothermia treatment paradigm (2, 29). For example, the subgroup analyses of a multicenter clinical trial revealed that therapeutic hypothermia resulted in better outcomes in TBI patients with subdural hematoma (compared with normothermia); whereas, the treatment was detrimental in patients with diffuse brain injury (30). Additionally, clinical and experimental studies demonstrated the neuroprotective benefits of slow and gradual rewarming over rapid rewarming in TBI (11, 31, 32). Although our SBC paradigm has been successful in many aspects (19–22), ameliorating cognitive deficits might require longer cooling duration (>8 h), a slower rewarming, or a lower temperature.

Subacute neurodegeneration and an exacerbated glial response were prominent following PBBI. However, they were not mitigated by the SBC treatment in the current study. Brain cooling targets multiple injury processes including excitotoxicity, apoptosis, and neuroinflammation (3) which occur mostly during the acute phase of TBI. Post-traumatic hypothermia has been shown to attenuate acute inflammatory response (at 4 and 24 h post-TBI) indicated by the altered M1/M2 phenotype balance in microglia, yet such an effect did not last beyond 7 days post-injury (16). Our previous study showed that 4or 8-h SBC reduced axonal injury, astrocytic and microglial activation at 3 days following isolated PBBI. At 21 days post-injury, however, SBC did not significantly ameliorate neurodegeneration measured by silver staining (22). It is plausible that the effects of SBC on these injury processes do not last more than a few hours or days. The treatment might slow down the abovementioned injury processes in the acute phase but not stop them from progressing in the later phase. Extending the cooling duration beyond the acute phase might improve long-term outcomes. Clinically, TBI patients are often cooled for a period of 24 h, up to several days, after injury with the core temperature targeted at 33–36°C (33). Yet it is both technically and logistically challenging to reproduce these clinical paradigms in the laboratory. Despite the lack of neuroprotective effects on subacute neurodegeneration and neuroinflammation, our data suggested that SBC significantly improved motor function following TBI and polytrauma. Experimental studies revealed the lasting effects of hypothermia on enhancing neurogenesis, gliogenesis, angiogenesis and neural connectivity (34), that might be associated with improved functional outcomes. These aspects need to be investigated further.

SBC had no effects on the subacute serum/plasma levels of pro-inflammatory cytokine IL-1β and anti-inflammatory cytokine IL-10. TBI triggers a complex sequence of inflammatory processes that contributes to the pathogenesis. Concurrent secondary insults such as hemorrhagic shock after TBI have been found to shift the cytokine response to a more anti-inflammatory phenotype. These pro-inflammatory and anti-inflammatory responses often occur acutely after TBI and are resolved by 1 or 2 weeks post-injury (35). Preclinical studies suggested that therapeutic hypothermia conveys neuroprotection by suppressing inflammatory processes (7, 36), whereas clinical studies showed mixed findings. In adult TBI patients, cerebrospinal fluid (CSF) level of IL-1β was found to be significantly suppressed during hypothermia (37). Such effect, however, was not observed in pediatric TBI (38). Our data showed that SBC did not affect the systemic cytokine levels at the subacute phase of TBI, again suggesting that the effect of SBC did not last beyond the acute phase. Intriguingly, the serum IL-10 level trended higher in the control group compared to the SBC group. Augmented IL-10 response was detected in animals subjected to TBI and hemorrhagic shock (39), as well as TBI patients with polytrauma (40). This might indicate that the injury effect persisted in the control group while it was suppressed by SBC in the treatment group.

There are several limitations in our study. First, the histopathology and cytokines measurement were performed at only one end point, although multiple mechanisms operating in the early and late post-trauma phases may contribute to the outcomes. While our previous studies have demonstrated a reduced astrocyte and microglial reactivity at 3 days post-injury following a 4-h SBC treatment in rats subjected to isolated PBBI (20, 22), it is unclear whether earlier assessment in the current combined injury model would have revealed more or less robust effects on these outcomes. Another limitation is the rewarming phase. The rate of rewarming following therapeutic hypothermia was suggested to be an important factor in its success or failure. Slow progressive rewarming optimizes the benefits of cooling whereas rapid active rewarming aggravates tissue damage leading to worse outcomes after TBI (31, 33, 41). In the current study, the animals were allowed to rewarm spontaneously, and it typically takes about 30 min for the rat brain to return back to baseline temperature. A slower, controlled rewarming may be needed to maximize the therapeutic benefits of SBC, especially the long-term outcomes.

Overall, the present study has demonstrated a persistent beneficial effect of SBC on motor function in a rat model of combined PBBI, hypoxemic and hypotensive insults. In addition, SBC transiently augmented cardiovascular function which may in part account for motor improvement. Although the treatment did not mitigate cognitive deficits and long-term histopathology, the findings underscore the compelling neuroprotective effect of SBC on motor deficits induced by TBI complicated by hypoxemic and hypotensive insults.

## Author contributions

LL wrote the manuscript and designed the study. LL, KC, XY, and BS contribute to data collection, data analysis or interpretation. JG and DS reviewed the study design and data analysis, and edited the manuscript.

### Conflict of interest statement

The authors declare that the research was conducted in the absence of any commercial or financial relationships that could be construed as a potential conflict of interest.
